# Supplementary material for: Tocotrienol-Rich Vitamin E (Tocovid) Improved Nerve Conduction Velocity in Type 2 Diabetes Mellitus Patients in a Phase II Double-Blind, Randomized Controlled Clinical Trial
Source: Nutrients. 2021 Oct 25;13(11):3770. doi: 10.3390/nu13113770 (PMC8618591; doi:10.3390/nu13113770)
Supplement: Supplementary file 1 [file nutrients-13-03770-s001.zip › Supplementary Materials_Table S1-S12.pdf]

nutrients

Supplementary Materials

**Table S1**	Glycemic control over 12 months by intervention group.
**Table S2**	Adverse events and serious adverse events reported by intervention group.
**Table S3**	Within group comparison of median nerve conduction parameters from baseline.
**Table S4**	Within group comparison of sural nerve conduction parameters from baseline.
**Table S5**	Within group comparison of tibial nerve conduction parameters from baseline.
**Table S6**	Compliance over 12 months by intervention group.
**Table S7**	Plasma vitamin E levels over 12 months by intervention group.
**Table S8**	Lipid profile over six months by intervention group.
**Table S9**	Between group comparison of changes in nerve conduction parameters in the low baseline CV subgroup.
**Table S10**	Between group comparison of changes in nerve conduction parameters in the high baseline CV subgroup.
**Table S11**	Lipid profile after six months washout by intervention group.
**Table S12**	Plasma vitamin E levels after six months washout by intervention group.

Table S1. Glycemic control over 12 months by intervention group.

HbA1c (%)	Tocovid (n = 42)		Placebo (n = 43)		*p*-Value (between groups) ‡
	Mean ± SD / Median (IQR)	p-value (within group) †	Mean ± SD / Median (IQR)	p-value (within group) †	
Baseline	7.53 ± 1.00 / 7.38 (1.50)		7.75 ± 1.16 / 7.75 (1.75)		0.363 ¶
At 2 months	7.52 ± 1.12	0.910	7.85 ± 1.28	0.410	
At 6 months	7.25 ± 1.15	0.111	7.66 ± 1.30	0.569	
At 12 months	7.34 (1.2)	0.500 §	8 (1.91)	0.200 §	
2 months changes	0 (0.78)		0.15 (0.9)		0.212
6 months changes	-0.1 (1.49)		-0.05 (0.88)		0.574
12 months changes	0.08 (1.11)		0.1 (1.35)		0.192

† *p*-value obtained from paired t-test test by comparing the mean within each intervention group. ‡ *p*-value obtained from Mann-Whitney U test. § *p*-value obtained from Wilcoxon signed rank test by comparing the median within each intervention group. ¶ *p*-value obtained from independent t-test.

Table S2. Adverse events and serious adverse events reported by intervention group.

Event	Tocovid (n=43)		Placebo (n=45)	
	Participants, n (%)	Events	Participants, n (%)	Events
Patients reporting more than one adverse event	10 (23.3)	-	9 (20)	-
Patients reporting one adverse event	12 (27.9)	-	14 (31.1)	-
Patients reporting a serious adverse event	4 (9.3)	-	1 (2.2)	-
**Adverse events (AEs)**				
Gastrointestinal disorders	5 (11.6)	5	5 (11.1)	6
Infections and infestations	5 (11.6)	6	2 (4.4)	2
Musculoskeletal disorders	5 (11.6)	5	3 (6.7)	3
Neurological disorders	4 (9.3)	6	1 (2.2)	1
Hypoglycemia	3 (7)	4	2 (4.4)	2
Lethargy and sleepiness	2 (4.7)	2	3 (6.7)	4
Dizziness	3 (7)	3	2 (4.4)	2
Dermatological disorders	3 (7)	3	2 (4.4)	2
Eye disorders	2 (4.7)	2	3 (6.7)	3
Psychiatric disorders	1 (2.3)	2	2 (4.4)	2
Respiratory disorders	1 (2.3)	1	2 (4.4)	2
Oedema	1 (2.3)	1	1 (2.2)	2
Urinary disorders	1 (2.3)	1	1 (2.2)	1
Non traumatic injury	0	0	1 (2.2)	1
Hematological disorders	0	0	1 (2.2)	1
Total AEs (%)		41 (54.7)		34 (45.3)
**Serious adverse events (SAEs)**				
Renal and urinary disorders	1 (2.3)	1	1 (2.2)	1
Mediastinal disorders	1 (2.3)	1	0	0
Uterine disorders	0	0	1 (2.2)	1
Viral fever	1 (2.3)	1	0	0
Total SAEs (%)		3 (60)		2 (40)

Table S3. Within group comparison of median nerve conduction parameters from baseline.

Median Sensory Nerve	Tocovid (n=68)			Placebo (n=65)		
	Mean ± SD / Median (IQR)	Difference from baseline M/MD (95% CI)	*p*-Value †	Mean ± SD / Median (IQR)	Difference from baseline M/MD (95% CI)	*p*-Value †
NP Amplitude (μV)						
At baseline	19.5 (14.0)	-	-	17.5 (12.0)		-
At 2 months	22.0 (16.4)	1.40 (0.05, 2.75)	0.040 *	17.3 (11.9)	0.82 (-0.35, 2.00)	0.155
At 6 months	22.5 (16.4)	1.20 (0.05, 2.60)	0.036 *	17.0 (12.3)	0.85 (-0.35, 2.20)	0.149
At 12 months	20.8 (15.5)	0.25 (-0.95, 1.54)	0.680	17.1 (15.2)	0.37 (-1.15, 1.85)	0.630
PP Amplitude (μV)						
At baseline	31.9 (22.7)	-	-	27.3 (14.7)		-
At 2 months	33.8 (22.0)	2.90 (0.60, 5.35)	0.013 *	28.8 (19.9)	0.80 (-0.85, 2.45)	0.308
At 6 months	38.8 (20.8)	3.25 (0.60, 6.45)	0.016 *	29.3 (17.8)	1.05 (-0.85, 2.80)	0.247
At 12 months	36.7 (20.8)	3.35 (-0.05, 5.95)	0.051	28.6 (18.3)	1.20 (-1.75, 3.75)	0.401
Conduction Velocity (m/s)						
At baseline	44.0 ± 6.79 / 42.8 (9.4)	-	-	43.2 ± 7.10 / 43.8 (9.10)	-	-
At 2 months	44.8 (11.9)	2.00 (1.40, 2.60)	<0.001***	42.7 ± 6.36	-0.42 (-1.06, 0.23)	0.204 ‡
At 6 months	43.9 ± 7.39	-0.17 (-1.55, 1.22)	0.810 ‡	42.6 ± 6.20	-0.52 (-1.11, 0.08)	0.090 ‡
At 12 months	46.4 (9.17)	2.08 (1.26, 2.90)	<0.001 ***	44.9 (6.10)	0.70 (-0.30, 1.70)	0.153
Peak Velocity (m/s)						
At baseline	35.2 ± 4.80 / 34.1 (6.0)	-	-	34.7 ± 5.06	-	-
At 2 months	35.2 (7.95)	1.05 (0.60, 1.60)	<0.001 ***	36.2 ± 4.81	-0.34 (-0.82, 0.13)	0.152 ‡
At 6 months	35.9 ± 4.58	0.71 (0.19, 1.24)	0.009 **	34.3 ± 4.45	-0.44 (-1.09, 0.20)	0.177 ‡
At 12 months	34.1 (7.46)	0.45 (-0.35, 1.10)	0.296	34.8 ± 4.26	0.07 (-0.64, 0.78)	0.851 ‡

* $p < 0.05$, ** $p < 0.01$, *** $p < 0.001$

† *p*-value obtained from Wilcoxon signed rank test by comparing the median within each intervention group. ‡ *p*-value obtained from paired t-test test by comparing the mean within each intervention group.

Abbreviations: M: Mean; NP: Negative peak; PP: Peak to peak.

Table S4. Within group comparison of sural nerve conduction parameters from baseline.

Sural Sensory Nerve	Tocovid (n=55)			Placebo (n=45)		
	**Mean ± SD / Median (IQR)**	**Difference from baseline M/MD (95% CI)**	***p*-Value †**	**Mean ± SD / Median (IQR)**	**Difference from baseline M/MD (95% CI)**	***p*-Value †**
NP Amplitude (μV)						
At baseline	9.7 (8.00)	-	-	10.1 (4.9)		-
At 2 months	8.5 (7.75)	0.20 (-0.75, 1.15)	0.611	8.5 (5.3)	-0.30 (-1.10, 0.30)	0.304
At 6 months	9.3 (6.4)	0.55 (-0.55, 1.45)	0.260	7.8 (6.1)	-0.85 (-1.80, 0.25)	0.153
At 12 months	10.7 (4.35)	-0.35 (-1.75, 1.23)	0.709	8.8 (5.58)	-0.60 (-1.65, 0.65)	0.326
PP Amplitude (μV)						
At baseline	6.6 (8.3)	-	-	8.7 (7.8)		-
At 2 months	6.5 (9.0)	1.00 (0.35, 1.70)	0.003 **	7.4 (8.2)	0.40 (-0.51, 1.30)	0.378
At 6 months	7.8 (10.8)	2.25 (1.15, 3.65)	<0.001 ***	9.4 (6.7)	0.60 (-0.60, 2.00)	0.310
At 12 months	8.32 (6.85)	2.10 (0.75, 3.40)	0.004 **	8.2 (8.2)	1.17 (-0.30, 2.40)	0.111
Conduction Velocity (m/s)						
At baseline	43.8 (7.75)	-	-	43.0 ± 4.98	-	-
At 2 months	44.4 (7.80)	1.75 (1.40, 2.20)	<0.001 ***	42.3 ± 4.51	-0.69 (-1.22, -0.16)	0.012 *‡
At 6 months	44.4 (8.65)	1.60 (1.10, 2.30)	<0.001 ***	42.2 ± 4.68	-0.74 (-1.23, -0.24)	0.005 **‡
At 12 months	44.4 (8.25)	1.75 (0.95, 2.39)	<0.001 ***	43.5 ± 4.43	0.51 (-0.35, 1.38)	0.236 ‡
Peak Velocity (m/s)						
At baseline	34.1 (7.05)	-	-	34.4 ± 3.7 / 33.7 (5.1)	-	-
At 2 months	34.6 (7.15)	1.15 (0.80, 1.60)	<0.001 ***	33.3 (3.20)	-0.65 (-1.15, -0.05)	0.041 *
At 6 months	35.0 (6.05)	1.40 (0.85, 1.95)	<0.001 ***	34.3 ± 3.6	-0.04 (-0.55, 0.46)	0.860 ‡
At 12 months	38.4 (7.50)	3.00 (2.10, 4.54)	<0.001 ***	35.3 ± 3.93	0.89 (0.12, 1.66)	0.024 *‡

* $p < 0.05$, ** $p < 0.01$, *** $p < 0.001$

† *p*-value obtained from Wilcoxon signed rank test by comparing the median within each intervention group. ‡ *p*-value obtained from paired t-test test by comparing the mean within each intervention group.

Abbreviations: M: Mean; NP: Negative peak; PP: Peak to peak.

Table S5. Within group comparison of tibial nerve conduction parameters from baseline.

Tibial Motor Nerve	Tocovid (n=76)			Placebo (n=73)		
	**Mean ± SD / Median (IQR)**	**Difference from baseline M/MD (95% CI)**	***p*-Value †**	**Mean ± SD / Median (IQR)**	**Difference from baseline M/MD (95% CI)**	***p*-Value †**
Conduction Velocity (m/s)						
At baseline	41.5 ± 5.54	-	-	39.9 ± 6.15 / 40.5 (10.5)	-	-
At 2 months	42.4 ± 4.83	0.88 (0.31, 1.45)	0.003 **‡	38.7 ± 5.82	-1.15 (-1.86, -0.45)	0.002 **‡
At 6 months	42.4 ± 5.10	0.93 (0.19, 1.67)	0.014 *‡	39.5 ± 5.29	-0.37 (-1.36, 0.62)	0.463 ‡
At 12 months	41.8 ± 5.38	0.27 (-0.64, 1.18)	0.555 ‡	39.6 (8.3)	-1.25 (-2.20, -0.20)	0.023 *
Distal Amplitude at Ankle (mV)						
At baseline	7.25 (5.75)	-	-	7.6 (7.2)	-	-
At 2 months	8.8 (5.98)	1.05 (0.70, 1.45)	<0.001 ***	8.9 (6.3)	0.75 (0.45, 1.15)	<0.001 ***
At 6 months	8.23 (5.83)	0.60 (0.05, 1.20)	0.041 *	8.7 (6.1)	0.75 (0.40, 1.15)	<0.001 ***
At 12 months	9.56 (4.40)	1.37 (0.73, 2.00)	<0.001 ***	9.3 (6.6)	0.65 (0.25, 1.10)	0.001 **
Proximal Amplitude at Knee (mV)						
At baseline	5.20 (3.98)	-	-	5.5 (5.90)	-	-
At 2 months	6.2 (4.35)	0.70 (0.45, 1.00)	<0.001 ***	5.8 (5.6)	0.65 (0.35, 0.90)	<0.001 ***
At 6 months	6.15 (4.5)	0.50 (0.25, 0.85)	<0.001 ***	6.3 (5.6)	0.65 (0.35, 1.00)	<0.001 ***
At 12 months	6 (4.26)	0.55 (0.13, 0.95)	0.013 *	5.5 (5.2)	0.35 (-1.78×10^{-5}, 0.70)	0.056

* $p < 0.05$, ** $p < 0.01$, *** $p < 0.001$

† *p*-value obtained from Wilcoxon signed rank test by comparing the median within each intervention group. ‡ *p*-value obtained from paired t-test test by comparing the mean within each intervention group.

Table S6. Compliance over 12 months by intervention group.

Compliance	Tocovid (n = 42)	Placebo (n = 43)	*p*-Value†
Baseline to 2 months	97.9 (5.69)	98.2 (8.94)	0.895
2 to 6 months	97.8 (9.36)	97 (7.49)	0.801
6 to 12 months	97 (5.9)	97 (4.33)	0.912
Over 12 months	96.6 (6.84)	96.1 (6.90)	0.626

Data presented as median (interquartile range).

† *p*-Value obtained from Mann-Whitney test.

Table S7. Plasma vitamin E levels over 12 months by intervention group.

Plasma vitamin E levels	Tocovid (n = 42)		Placebo (n = 43)		*p*-Value (between groups) ‡
		p-Value (within group) †		*p*-Value (within group) †	
α-Tocotrienol (pg/mL)					
At baseline	37.3 (55.1)		37.5 (48.8)		0.629
At 2 months	67.6 (109)	0.007 **	20.9 (44.6)	0.329	
At 6 months	59.5 (238)	0.039 *	18.0 (54.4)	0.611	
At 12 months	50.9 (35.7)	0.701	22.5 (21.1)	<0.001 ***	
2 months changes	29.2 (99.7)		-8.01 (26.5)		0.005 **
6 months changes	16.5 (152)		-13.3 (72.5)		0.051
12 months changes	10.4 (77.2)		-13.4 (26.3)		0.012 *
γ-Tocotrienol (pg/mL)					
At baseline	22.3 (31.0)		24.7 (28.5)		0.913
At 2 months	74.7 (142)	0.003 **	7.46 (16.9)	<0.001 ***	
At 6 months	31.28 (133)	0.007 **	11.6 (21.0)	0.002 **	
At 12 months	30.0 (28.7)	0.472	13.2 (17.9)	0.004 **	
2 months changes	38.7 (114)		-10.4 (22.0)		<0.001 ***
6 months changes	12.1 (114)		-10.1 (22.0)		<0.001 ***
12 months changes	5.82 (34.8)		-13.5 (35.6)		0.013 *
δ-Tocotrienol (pg/mL)					
At baseline	12.7 (16.2)		12.7 (13.6)		0.776
At 2 months	32.2 (71.7)	<0.001 ***	4.4 (11.2)	0.037 *	
At 6 months	13.7 (50.5)	0.072	7.41 (9.79)	0.033 *	
At 12 months	12.7 (7.68)	0.446	5.39 (5.77)	<0.001 ***	
2 months changes	12.4 (50.1)		-4.92 (10.6)		<0.001 ***
6 months changes	5.09 (44.6)		-3.41 (9.75)		0.011 *
12 months changes	0.87 (17.6)		-6.45 (12.4)		0.014 *
α-Tocopherol (ng/mL)					
At baseline	1.64 (1.22)		1.92 (1.57)		0.861
At 2 months	1.60 (2.97)	0.339	1.07 (1.02)	0.033 *	
At 6 months	1.27 (5.45)	0.457	0.80 (0.76)	0.034 *	
At 12 months	1.28 (0.30)	0.003 **	0.97 (0.46)	<0.001 ***	
2 months changes	-0.02 (1.60)		-0.48 (1.12)		0.005 **
6 months changes	-0.06 (2.23)		-0.69 (1.14)		0.006 **
12 months changes	-0.29 (1.47)		-0.87 (1.10)		0.100

* $p < 0.05$, ** $p < 0.01$, *** $p < 0.001$

Data presented as median (interquartile range).

† *p*-value obtained from Wilcoxon signed rank test by comparing the median within each intervention group. ‡ *p*-value obtained from Mann-Whitney U test by comparing the median between Tocovid and placebo groups.

Table S8. Lipid profile over six months by intervention group.

Lipid profile	Tocovid (n = 42)		Placebo (n = 43)		*p*-Value (between groups) ‡
		p-Value (within group) †		*p*-Value (within group) †	
TC (mmol/L)					
At baseline	4.88 ± 1.08 / 4.80 (1.36)		4.46 ± 1.00 / 4.22 (1.25)		0.067 ¶
At 2 months	4.93 ± 0.99	0.607 §	4.17 (1.47)	0.305	
At 6 months	4.57 (1.30)	0.196	4.2 (0.8)	0.190	
2 months changes	-0.11 (0.91)		-0.10 (0.79)		0.601
6 months changes	-0.26 (0.96)		-0.03 (0.67)		0.758
TG (mmol/L)					
At baseline	1.47 (0.92)		1.53 (1.05)		0.610
At 2 months	1.60 (1.01)	0.368	1.63 (0.89)	0.740	
At 6 months	1.49 (0.95)	0.565	1.44 (0.85)	0.145	
2 months changes	0.04 (0.94)		-0.09 (0.74)		0.298
6 months changes	0.06 (0.89)		-0.03 (0.62)		0.210
HDL-C (mmol/L)					
At baseline	1.3 (0.4) / 1.32 ± 0.27		1.20 (0.44)		0.266
At 2 months	1.30 (0.48)	0.485	1.3 (0.4)	0.357	
At 6 months	1.42 ± 0.33	0.004 **§	1.30 (0.35)	<0.001 ***	
2 months changes	0.03 ± 0.24		0.02 ± 0.22		0.891 ¶
6 months changes	0.1 (0.2)		0.1 (0.2)		0.814
LDL-C (mmol/L)					
At baseline	2.60 (1.55) / 2.77 ± 1.03		2.30 (1.15)		0.099
At 2 months	2.80 ± 0.91	0.749 §	2.30 (1.15)	0.250	
At 6 months	2.37 (0.98)	0.017 *	2.0 (0.7)	0.009 **	
2 months changes	0.10 (0.78)		-0.10 (0.65)		0.240
6 months changes	-0.30 (0.88)		-0.10 (0.63)		0.823

* $p < 0.05$, ** $p < 0.01$, *** $p < 0.001$

Data presented as median (interquartile range) or mean ± standard deviation.

† *p*-value obtained from Wilcoxon signed rank test by comparing the median within each intervention group. ‡ *p*-value obtained from Mann-Whitney U test by comparing the median between Tocovid and placebo groups. § *p*-value obtained from paired t-test by comparing mean within each intervention group. ¶ *p*-value obtained from independent t-test by comparing the mean between Tocovid and placebo groups.

Abbreviations: TC: total cholesterol; TG: triglycerides; HDL-C: high-density lipoprotein cholesterol; LDL-C: low-density lipoprotein cholesterol.

Table S9. Between group comparison of changes in nerve conduction parameters in the low baseline CV subgroup.

Baseline characteristics	Tocovid (n)	Placebo (n)	2 months changes			6 months changes			12 months changes		
			**Tocovid**	**Placebo**	***p*-Value †**	**Tocovid**	**Placebo**	***p*-Value †**	**Tocovid**	**Placebo**	***p*-Value †**
Median CV < 43.61 m/s [a] (N=68)											
NPA			0.85 (4.60)	-0.05 (4.08)	0.258	0.30 (3.80)	0.60 (4.58)	0.619	0.98 (5.06)	0.90 (6.01)	0.949
PPA	36	32	1.1 (8.6)	0.10 (6.25)	0.326	3.95 (13.55)	0.90 (7.35)	0.206	3.1 (17.1)	4.9 (9.8)	0.956
CV			1.60 (2.25)	0.20 (2.88)	0.008 **	1.75 (2.13)	0.20 (3.25)	0.096	2.95 (4.42)	2.55 (3.03)	0.370
PV			1.07 ± 1.47	0.32 ± 1.84	0.067 ‡	0.95 (2.20)	0 (2.93)	0.037 *	0.67 ± 2.24	1.37 ± 2.23	0.203 ‡
Sural CV < 43.16 m/s [a] (N=51)											
NPA			0.45 ± 2.02	-0.62 ± 2.66	0.111 ‡	0.36 ± 2.90	-1.58 ± 3.13	0.026 *‡	1.82 ± 3.55	-0.99 ± 3.83	0.009 **‡
PPA	26	25	0.79 ± 2.12	0.19 ± 2.65	0.380 ‡	1.70 (4.85)	-0.5 (4.5)	0.070	3.74 (5.12)	1.0 (5.5)	0.014 *
CV			1.85 (1.28)	0.0 (1.3)	<0.001 ***	1.7 (2.3)	0 (0.7)	<0.001 ***	2.95 ± 3.23	1.59 ± 2.55	0.102 ‡
PV			1.35 (1.48)	0.0 (1.5)	<0.001 ***	2.29 ± 1.79	0.43 ± 1.73	<0.001 ***‡	3.20 (6.84)	0.4 (3.4)	<0.001 ***
Tibial CV < 40.71 m/s [a] (N=78)											
CV			1.05(2.50)	-0.3 (1.9)	<0.001 ***	0.85 (3.20)	0 (1.83)	0.015 *	1.78 ± 4.27	0.39 ± 4.21	0.152 ‡
AA	38	40	0.80 (1.88)	0.40 (1.25)	0.276	0.00 (2.75)	0.55 (1.48)	0.291	1.95 (3.18)	0.25 (1.37)	0.016 *
KA			0.55 (1.20)	0.50 (1.43)	0.869	0.55 (1.88)	0.70 (1.55)	0.453	0.40 (1.71)	0.15 (1.43)	0.217

* $p < 0.05$, ** $p < 0.01$, *** $p < 0.001$

Data presented as median (interquartile range) or mean ± standard deviation.

[a] Mean CV; † *p*-value obtained from Mann-Whitney U test by comparing the median between Tocovid and placebo groups. ‡ *p*-value obtained from independent t-test by comparing the mean between Tocovid and placebo groups.

Abbreviations: n: Number of participants in each intervention group; N: Number of participants in each subgroup for each nerve.

Table S10. Between group comparison of changes in nerve conduction parameters in the high baseline CV subgroup.

Baseline characteristics	Tocovid (n)	Placebo (n)	2 months changes			6 months changes			12 months changes		
			Tocovid	Placebo	*p*-Value	Tocovid	Placebo	*p*-Value	Tocovid	Placebo	*p*-Value
Median CV ≥ 43.61 m/s [a] (N=65)											
NPA	32	33	1.28 ± 7.34	1.81 ± 4.91	0.737	1.30 (7.15)	0.5 (6.7)	0.694	-0.35 (5.78)	0.9 (8.7)	0.808
PPA			3.46 ± 10.4	1.31 ± 8.02	0.353	1.85 (13.85)	0.6 (7.0)	0.632	4.05 (10.75)	1.1 (12.8)	0.194
CV			0.95 (3.00)	-0.7 (3.10)	<0.001 ***	0.35 (2.95)	-1.2 (2.3)	0.008 **	0.9 (3.30)	-0.8 (2.3)	0.005 **
PV			0.80 (2.95)	-0.5 (1.4)	<0.001 ***	0.26 ± 2.30	-1.22 ± 1.67	0.004 **	0.55 (4.20)	-0.5 (2.3)	0.203
Sural CV ≥ 43.16 m/s [a] (N=49)											
NPA	29	20	0.1 (5.7)	-0.25 (1.95)	0.684	0.3 (4.8)	0.00 (5.65)	0.927	-3.12 ± 6.09	0.18 ± 4.83	0.049 *
PPA			1.40 ± 2.76	0.73 ± 3.41	0.453	2.40 ± 4.07	1.59 ± 4.84	0.533	0.70 (4.06)	2.20 (2.79)	0.422
CV			1.23 ± 1.70	-1.80 ± 1.47	<0.001 ***	0.91 ± 2.13	-1.50 ± 1.53	<0.001 ***	1.0 (3.3)	-0.65 (2.63)	0.065
PV			0.79 ± 1.59	-1.36 ± 1.56	<0.001 ***	0.53 ± 1.76	-0.64 ± 1.44	0.019 *	2.12 ± 2.70	0.30 ± 2.64	0.023 *
Tibial CV ≥ 40.71 m/s [a] (N=71)											
CV	38	33	0.40 (1.45)	-2.5 (3.0)	<0.001 ***	-0.35 ± 2.31	-2.21 ± 2.62	0.002 **	-2.00 (3.18)	-3.30 (4.18)	0.032 *
AA			0.80 (1.78)	0.9 (2.0)	0.716	0.85 (2.35)	0.9 (2.2)	0.608	0.98 ± 2.34	1.46 ± 2.68	0.426
KA			0.65 (1.33)	0.5 (1.4)	0.645	0.40 (1.45)	0.1 (1.4)	0.482	0.23 (2.48)	0.3 (2.1)	0.769

* $p < 0.05$, ** $p < 0.01$, *** $p < 0.001$

Data presented as median (interquartile range) or mean ± standard deviation.

[a] Mean CV; † *p*-value obtained from Mann-Whitney U test by comparing the median between Tocovid and placebo groups. ‡ *p*-value obtained from independent t-test by comparing the mean between Tocovid and placebo groups.

Abbreviations: n: Number of participants in each intervention group; N: Number of participants in each subgroup for each nerve.

Table S11. Lipid profile after six months washout by intervention group.

Lipid profile	Tocovid (n = 33)		Placebo (n = 39)		*p*-Value (between groups) ‡
		p-Value (within group) †		*p*-Value (within group) †	
**TC (mmol/L)**					
At baseline (B)	4.51 (1.19) / 4.66 ± 0.97		4.27 (1.25)		0.296
At washout (WO)	4.58 (1.14) / 4.63 ± 0.97	0.816 §	4.33 (1.29)	0.629	0.351
WO - B	0.03 ± 0.79		-0.02 ± 0.76		0.795 ¶
**TG (mmol/L)**					
At baseline (B)	1.27 (0.87)		1.53 (1.08)		0.224
At washout (WO)	1.37 (0.85)	0.710	1.37 (1.11)	0.214	0.505
WO - B	0 (0.68)		-0.05 (0.63)		0.261
**HDL-C (mmol/L)**					
At baseline (B)	1.4 (0.4) / 1.40 ± 0.34		1.2 (0.34)		0.079
At washout (WO)	1.30 (0.36) / 1.36 ± 0.26	0.347 §	1.27 (0.26)	**0.021** *	0.626
WO - B	0 (0.33)		0.06 (0.25)		0.288
**LDL-C (mmol/L)**					
At baseline (B)	2.5 (1.1) / 2.54 ± 0.84		2.30 (1.15)		0.309
At washout (WO)	2.46 (1.07) / 2.60 ± 0.89	0.586 §	2.11 (1.05)	0.194	0.175
WO - B	-0.06 ± 0.64		-0.15 ± 0.83		0.614 ¶

* $p < 0.05$, ** $p < 0.01$, *** $p < 0.001$

Data presented as median (interquartile range) or mean ± standard deviation.

† *p*-value obtained from Wilcoxon signed rank test by comparing the median within each intervention group. ‡ *p*-value obtained from Mann-Whitney U test by comparing the median between Tocovid and placebo groups. § *p*-value obtained from paired t-test by comparing mean within each intervention group. ¶ *p*-value obtained from independent t-test by comparing the mean between Tocovid and placebo groups.

Abbreviations: TC: total cholesterol; TG: triglycerides; HDL-C: high-density lipoprotein cholesterol; LDL-C: low-density lipoprotein cholesterol.

Table S12. Plasma vitamin E levels after six months washout by intervention group.

Plasma vitamin E levels	Tocovid (n = 33)		Placebo (n = 39)		*p*-Value (between groups) ‡
		p-value (within group) †		*p*-value (within group) †	
**α-Tocotrienol (pg/mL)**					
At baseline (B)	35.5 (30.9)		37.5 (48.8)		0.756
At 12 months (12M)	49.4 (27.4)	0.183	22.5 (19.9)	0.002 *	
At washout (WO)	12.5 (13.9)	0.012 *	9.56 (17.5)	<0.001 ***	0.148
WO – B	-11.7 (46.1)		-23.4 (40.2)		0.339
WO – 12M	-29.6 (33.7)		-8.17 (16.8)		0.002 **
**γ-Tocotrienol (pg/mL)**					
At baseline (B)	18.1 (13.3)		24.1 (29.4)		0.493
At 12 months (12M)	23.6 (24.1)	0.171	13.4 (17.7)	0.011 *	
At washout (WO)	5.76 (7.07)	<0.001 ***	4.03 (8.50)	<0.001 ***	0.044 *
WO – B	-12.1 (20.7)		-20.7 (32.6)		0.362
WO – 12M	-13.6 (29.9)		-7.68 (14.4)		0.049 *
**δ-Tocotrienol (pg/mL)**					
At baseline (B)	11.3 (9.76)		12.7 (12.6)		0.551
At 12 months (12M)	12.4 (10.3)	0.708	5.52 (5.77)	<0.001 ***	
At washout (WO)	3.15 (2.35)	<0.001 ***	2.73 (1.45)	<0.001 ***	0.047 *
WO – B	-8.25 (11.2)		-8.89 (12.5)		0.334
WO – 12M	-9.52 ± 7.43		-3.29 ± 5.01		<0.001 *** §
**α-Tocopherol (ng/mL)**					
At baseline (B)	1.43 (1.00)		1.88 (1.49)		0.275
At 12 months (12M)	1.30 (0.22)	0.221	0.98 (0.42)	<0.001 ***	
At washout (WO)	0.86 ± 0.27 / 0.83 (0.25)	<0.001 ***	0.93 ± 0.30 / 0.89 (0.44)	<0.001 ***	0.285 §
WO – B	-0.55 (0.88)		-0.84 (1.58)		0.559
WO – 12M	-0.49 (0.26)		-0.08 (0.33)		<0.001 ***

* $p < 0.05$, ** $p < 0.01$, *** $p < 0.001$

Data presented as median (interquartile range) or mean ± standard deviation.

† *p*-value obtained from Wilcoxon signed rank test by comparing the median within each intervention group. ‡ *p*-value obtained from Mann-Whitney U test by comparing the median between Tocovid and placebo groups. § *p*-value obtained from independent t-test by comparing the mean between Tocovid and placebo groups.
