# Supplementary material for: Tocotrienol-Rich Vitamin E (Tocovid) Improved Nerve Conduction Velocity in Type 2 Diabetes Mellitus Patients in a Phase II Double-Blind, Randomized Controlled Clinical Trial
Source: Nutrients. 2021 Oct 25;13(11):3770. doi: 10.3390/nu13113770 (PMC8618591; doi:10.3390/nu13113770)
Supplement: Supplementary file 1 [file nutrients-13-03770-s001.zip › Supplementary Materials_Vitamin E (Tocovid) constituents.pdf]

Constituents of Tocovid Suprabio^{TM}

Participants in the treatment group receive 200 mg of tocotrienol-rich vitamin E (Tocovid Suprabio^{TM}) twice daily. Each 200 mg soft capsule contains the following ingredients:

1. d-Alpha-Tocotrienol 61.52mg
2. d-Gamma-Tocotrienol 112.80mg
3. d-Delta-Tocotrienol 25.68mg
4. d-Alpha-Tocopherol 91.60IU
5. Plant Squalene 51.28mg
6. Phytosterol Complex 20.48mg
7. Phytocarotenoid Complex 360.00ug
